# Supplementary material for: Contribution of Morphoanatomic Characters to the Taxonomy of the Genus LAELIA (Orchidaceae) in Mexico and Their Implication in Environmental Adaptation
Source: Plants (Basel). 2023 Mar 1;12(5):1089. doi: 10.3390/plants12051089 (PMC10005411; doi:10.3390/plants12051089)
Supplement: Supplementary file 1 [file plants-12-01089-s001.zip › plants-2101696-supplementary.pdf]

Supplementary Materials.

List S1. Morphological characters based on Halbinger & Soto, 1997.

1. Flower arrangement. 0: Helically; 1: Sub-distically; 2: Solitary.
2. Arrangement of flowers. 0: In a compact sub-umbellate raceme; 1: In a loose raceme.
3. Column wings. 0: Basal triangular wingless in lateral view; 1: Wingless basal triangular in lateral view.
4. Peduncle bracts. 0: Much shorter than the internodes; 1: Above the half the length of the internode; 2: Subequal to internode.
5. Flower Bracts (extended / adpressed). 0: Extended; 1: Adpressed.
6. Callus of the lip. 0: With a basal part as a plate; 1: Without a basal part as a plate.
7. Colour of floral bracts. 0: Coloured like the tepals; 1: Not coloured like the tepals.
8. Colour of flowers. 0: Purple; 1: Yellow; 2: Pink to white.
9. Colour of ovary. 0: Mainly coloured like tepals; 1: Bronzed green.
10. Column. 0: Strongly arched; 1: Slightly arched; 2: straight.
11. Cuniculus (Conspicuous / Inconspicuous). 0: Conspicuous > 2 mm deep; 1: Inconspicuous < 2 mm deep).
12. Flowers. 0: Always resupinate; 1: Resupinated or not.
13.Shape of floral bracts. 0: Scale-like; 1: Shorter than the ovary; 2: Sub-equal to ovary.
14. Shape of the anther layer. 0: Saddle-shaped; 1: Slightly lobed; 2: ovoid-cordate.
15. Shape of petals. 0: Basally cuneate; 1: Sub clawed.
16. Shape of lower pollinia. 0: Oblong square; 1: Triangular oblanceolate; 2: Linguliform caudicles.
17. Shape of the pseudobulb. 0: Fusiform; 1: Ellipsoid-ovoid; 2: Conical-ovoid; 3: Sub-globose; 4: discoid.
18. Shape of the margin of the petals. 0: Wavy; 1: Not wavy.
19. Shape of sepal margin. 0: Wavy; 1: Not wavy.
20. Lip throat.0: Without a large brown spot; 1: With a large brown spot.
21. Habitat. 0: Medium altitude: From 800 to 2000 m asl; 1: Lowland altitude: 500 m asl; 2: High Mountain altitude: 2000-3000 m asl.
22. Stigmatic lobes. 0: Not protruding at the lower margin; 1: Protruding at the lower margin.
23. Peduncle. 0: Elongated, much longer than subtended leaves; 1: Slightly longer than the leaves.
24. Presence of lip lines. 0: With purple branching lines on the throat; 1: No branching purple lines on the throat.
25. Presence of viscidium on the rostellum. 0: Without a well-defined viscidium; 1: With a viscidium well defined.
26. Keels of lip. 0: Wave-dented in lateral view; 1: Entire in lateral view; 2: Absent.
27. Rhizomes. 0: Elongated; 1: Short; 2: Abbreviated.
28. Surface of pseudobulb. 0: Sulcate; 1: More wrinkled.
29. Surface of the ovary. 0: Smooth; 1: Scaly.
30. Stigmatic surface. 0: Not hidden by the rostellum; 1: Hidden by the rostellum.

31. Size of petals. 0: As broad as the sepals; 1: Conspicuously wider than the sepals.
32. Blooming season. 0: In autumn; 1: In winter; 2: In spring.
33. Texture of lip. 0: Glabrous; 1: Pubescent.
34. Type of capsule. 0: Conspicuous keel, dark ridges, warty punctuation, type *Schomburgkia*; 1: Low keel, inconspicuous ridges, smooth, like *Laelia anceps*; 2: High keel, combs blunt, smooth, type *L. autumnalis*; 3: High keel, well defined ridges, smooth, type *L. speciosa*; 4: Narrow and low keel, dark ridges, minutely papillose, type *L. rubescens*; 5: Ellipsoid capsule.
35. Type of leaves. 0: Fleshy coriaceous; 1: Chartaceous coriaceous.
36. Type of inflorescence. 0: From the mature pseudobulb; 1: From the pseudobulb in developing.
37. Type of pseudobulb. 0: With stem; 1: Sessile.
38. Internodes in the inflorescence. 0: Less than 9 internodes; 1: Five to nine.
39. Number of leaves in the pseudobulb. 0: two to three; 1: One.
40. Number of internodes in the pseudobulb. 0: two; 1: three; 2: four; 3: More than 4.
41. Number of internodes in the rhizome. 0: four to six; 1: three to four.
42. Proportion of blade (length/width). 0: Under 4.2; 1:4.21 to 5.8; 2: 5.81 to 7.4; 3: Greater than 7.41.
43. Number of flowers by cluster. 0: one to two flowers; 1: three to five flowers; 2: More than 6 flowers.
44. Stigmatic surface (width/length ratio. 0: < 1.0; 1: > 1.1.

List S2. Anatomical characters.

Middle region of leaf blade:

1. Abundance of crystals in mesophyll. 0: Scarce; 1: Don´t apply.
2. Substomatal chamber. 0: Inconspicuous; 1: Conspicuous.
3. Bulliform cells in the central zone. 0: Absent; 1: Present.
4. Water cells in the mesophyll. 0: Absent; 1: Present.
5. Calcium oxalate crystals. 0: Absent; 1: Present.
6. Distribution of abaxial epidermal cells. 0: Rows; 1: Alternate.
7. Distribution of adaxial epidermal cells. 0: Rows; 1: Alternate.
8. Distribution of stomata in the abaxial epidermis. 0: rows; 1: alternate.
9. Distribution of the layers of the abaxial hypodermis. 0: Discontinuous; 1: continue; 2: Don´t apply.
10. Distribution of the layers of the adaxial hypodermis. 0: Discontinuous; 1: continuous.
11. Stegmatomas in non-vascular fiber bundles. 0: Absent; 1: Present,
12. Shape of the cells of the abaxial hypodermis. 0: Oblong; 1: Polygonal Isodiametric; 2: Elongated traverse; 3: Don´t apply.
13. Shape of the cells of the adaxial hypodermis. 0: Oblong; 1: Polygonal elongated; 2: Isodiametric traverse.
14. Shape of abaxial epidermal cells. 0: Elongated polygonal; 1: Polygonal isodiametric; 2: Oblong.

15. Shape of adaxial epidermal cells. 0: Elongated polygonal, 1: Polygonal isodiametric; 2: Oblong.
16. Shape of the crystals. 0: Raphides; 1: Does not apply; 2: Raphides and sand.
17. Thickness of the cell walls of the abaxial hypodermis. 0: Thin; 1: Gross.
18. Thickness of the cell walls of the adaxial hypodermis. 0: Thin. 1: Gross
19. Presence of abaxial hypodermis. 0: Absent; 1: Present.
20. Papillae in the abaxial epidermis. 0: Absent; 1: Present.
21. Position of occlusive cells with respect to the abaxial epidermis. 0: At the level; 1: Semi sunk; 2: Sunk.
22. Cells with secondary thickening in the mesophyll. 0: Present; 1: Absent.
23. Strata of fibre bundles. 0: Absent; 1: Present.
24. Cuticle surface texture. 0: Smooth; 1: Striated; 2: Granular; 3: Rough.
25. Type of development of the outer cuticular ridges of the stomata. 0: Light; 1: Deep.
26. Type of mesophile. 0: Homogeneous; 1: Heterogeneous.
27. Type of abaxial papillae. 0: Cuticular; 1: Epidermal; 2: Does not apply.
28. Type of cell wall in abaxial hypodermis. 0: Cellulosic; 1: Lignified; 2: Don´t apply.
29. Type of cell wall in adaxial hypodermis. 0: Lignified; 1: Cellulosic.
30. Type of parenchyma. 0: Palisade; 1: Spongy; 2: Both.
31. Types of vascular bundles. 0: Three; 1: Four; 2: Five.
32. Trichomes. 0: Absent; 1: Present.
33. Location of stomata. 0: In adaxial epidermis; 1: In abaxial epidermis; 2: In both epidermises.
34. Location of extravascular fibre bundles. 0: Throughout the mesophyll; 1: Abaxial and adaxial.
35. Location of the hypodermis. 0: Adaxial; 1: Abaxial; 2: Both.
36. Location of oxalate crystals of calcium. 0: Not applicable; 1: Centre of the mesophyll; 2: Hypodermis.
37. Wall thickness of extravascular fibres. 0: Thick 5µm; 1: Thin 2.5µm.
38. Number of layers of extravascular adaxial fiber bundles. 0: 1 to 2; 1: 2.1 to 3; 2: 3.1 to 4; 3: 4.1 to 5.
39. Number of layers of extravascular abaxial fiber bundles. 0: 2 to 4; 1: 4.1 to 6; 2: 6.1 to 9; 3: Does not apply.
40. Number of layers of vascular bundles. 0. One; 1; Two; 2. Three.
41. Number of vascular bundles (along the length of the lamina). 0: From 30 to 50 vascular bundles; 1: from 50 to 70; 2: from 71 to 90; 3: more than 90.
42. Number of bulliform cell layers.0: Two; 1: Three; 2: Four; 3: Five; 4: Does not apply.
43. Number of layers of adaxial hypodermis. 0: One; 1: Two; 2: Does not apply.
44. Number of layers of abaxial hypodermis. 0: One; 1: Two; 2: Does not apply.
45. Proportion of palisade parenchyma. 0: 1/3 of the mesophyll =0 to 40%; 1: 2/3 of the mesophyll=41 to 80%; 2:1 the whole of mesophyll= 81 to 100%.

Basal region of the leaf blade:

46. Airspaces. 0: Absent; 1: Present.
47. Hypodermis. 0: Absent; 1: Present.
48. Type of mesophyll. 0: Homogeneous; 1: Heterogeneous.
49. Type of parenchyma in mesophyll. 0: Palisade; 1: Spongy; 2: Both.
50. Location of the hypodermis. 0: Adaxial; 1: Abaxial; 2: Both.
51. Number of layers of adaxial hypodermis. 0: One; 1: Two or more; 2. Does not apply.
52. Number of layers of abaxial hypodermis. 0: One; 1: Two or more; 2: Does not apply.

List S3. Continuous quantitative anatomical characters

1. High abaxial epidermal cells, in cross-sectional view (µm). 0: Greater than 30; 1: Less than 30.
2. High adaxial epidermal cells, in cross-sectional view (µm). 0: 30 to 36; 1: 24 to 30; 2: 18 to 24.
3. Width of abaxial epidermal cells, in cross-sectional view (µm). 0: 25 to 29; 1: 29 to 3; 2: 33 to 37; 3: 37 to 41.
4. Width of adaxial epidermal cells, in cross sectional view (µm). 0: 23 to 27; 1: 27 to 31.
5. Area of abaxial cells (μm^2). 0: 600 to 800; 1: 801 to 1000; 2: 1001 to 1200; 3: 1201 to 1400.
6. Area of adaxial cells (μm^2). 0: Greater than 5000; 1: Less than 5000.
7. Abaxial cuticle thickness (µm). 0: Greater than 18; 1: Under 18.
8. Adaxial cuticle thickness (µm). 0: Greater than 12; 1: Less than 12.
9. Thickness of the mesophyll (µm). 0: 500 to 1000; 1: 1001 to 1500; 2: 1501 to 2000; 3: 2001 to 2500.
10. Stomatal index. 0: Greater than 7; 1: Less than 7.
11. Length of guard cells (µm). 0: Greater than 45; 1: 35 at 40 µm; 2: Less than 35 µm.
